# Supplementary material for: Enzymatic Inhibitors of Aspartyl Protease EAP1 and Xylanase SRXL1 from Sporisorium reilianum Isolated from Corn Seeds
Source: Int J Mol Sci. 2025 Oct 14;26(20):9974. doi: 10.3390/ijms26209974 (PMC12562423; doi:10.3390/ijms26209974)
Supplement: Supplementary file 1 [file ijms-26-09974-s001.zip › ijms-3806047-supplementary.pdf]

Supplementary material

Table S1. Percentage inhibition of the aspartyl protease EAP1 and xylanase SRXL1 enzymes of *S. reilianum* by fractions collected during the purification process of enzyme inhibitors derived from two corn varieties, determined using ion exchange chromatography.

Fraction	Inhibition percentage (%)			
	Corn hybrid DK-2061		Corn hybrid BOGUI	
	EAP1	SRXL1	EAP1	SRXL1
1-11	64.0±0.9	32.8±1.7	58.7±1.2	48.8±0.3
12-16	64.0±0.9	51.8±0.7	58.7±1.2	23.9±1.7
17-23	100.0±0.0	100.0±0.0	19.9±0.7	21.6±1.7
24-30	39.9±1.6	16.9±1.0	64.0±0.7	100.0±0.0
31-45	31.0±1.1	16.7±0.7	18.0±0.5	40.3±0.3
Control acetate buffer	00.0±0.0	00.0±0.0	00.0±0.0	00.0±0.0
Control acetate-NaCl buffer	00.0±0.0	00.0±0.0	00.0±0.0	00.0±0.0

The values represent the average of three replicates, along with their standard deviation.

Table S2. EDS-based elemental composition of the flours, aqueous extracts, and purified inhibitors with activity against the enzymes aspartyl protease EAP1 and xylanase SRXL1 of *S. reilianum*, obtained from the seeds of two corn hybrids.

Hybrid	Element	Atomic percentage		
		Flour	Aqueous extract	Purified inhibitor
DK-2061	O	24.8±1.0	30.0±1.1	28.7±0.9
	C	73.6±1.9	67.3±1.7	55.0±1.4
	N	1.6±0.1	0.3±0.16	0.5±0.1
	P	0.0±0.0	2.4±0.2	0.0±0.0
	Al	0.0±0.0	0.0±0.0	0.7±0.1
	Na	0.0±0.0	0.0±0.0	12.3±1.3
	Cl	0.0±0.0	0.0±0.0	2.8±0.6
	Total	100.00	100.00	100.00
BOGUI	O	27.2±1.1	30.8±1.2	16.5±0.6
	C	70.4±1.8	66.4±1.7	63.4±2.1
	N	2.4±0.2	2.4±0.2	0.0±0.0
	P	0.0±0.0	0.0±0.0	0.0±0.0
	Al	0.0±0.0	0.4±0.1	0.2±0.1
	Na	0.0±0.0	0.0±0.0	11.9±1.5
	Cl	0.0±0.0	0.0±0.0	7.9±1.8
	Total	100.00	100.00	100.00

The determinations were performed in triplicate, with five points sampled on the surface of the material. The values represent the average of the measurements.

Table S3. Qualitative assessment of starch in various samples with inhibitory activity against the enzymes aspartyl protease EAP1 and xylanase SRXL1 of *S. reilianum* obtained from corn grains.

Sample	Result
Water	-
Corn starch	+++
DK-2061 flour	++
BOGUI flour	++
DK-2061 aqueous extract	++
BOGUI aqueous extract	++
DK-2061purified inhibitor	+
BOGUI purified inhibitor	+

The number of + symbols indicates the intensity of the color in the reaction. The determinations were performed in triplicate.

Table S4. Statistical analysis of the data obtained from the inhibitory activity of aqueous extracts from two corn hybrid varieties (DK-2061 and BOGUI) on the aspartyl protease enzyme EAP1 of *S. reilianum*.

Test of the Normality Distribution

Test Name	Test Value	Prob Level	10% Critical Value	5% Critical Value	Decision (Alpha= 5%)
Kolmogorov-Smirnov	0.175		0.383	0.415	Can't reject normality

Test of the Homoscedasticity

Test Name	Test Value	Prob Level	Reject Equal Variances? (α=0.20)
Levene (Data – Medians)	1.7196	0.25995	No

Analysis of Variance Table and F-Test

Model Term	DF	Sum of Squares	Mean Square	F-Ratio	Prob Level	Reject Equal Means? (α=0.05)	Power (α=0.05)
Between (Corn)	1	54.65463	54.65463	10.6565	0.03095	Yes	0.68861
Within (Error)	4	20.51498	5.128746				
Adjusted Total	5	75.16961					
Total	6						

Tukey-Kramer Multiple-Comparison Test

Response: Porcentage_of_inhibition
Term A: (Corn)

Alpha=0.050 Error Term=S(A) DF=4 MSE=5.128746 Critical Value=3.9342

Group	Count	Mean	Different From Groups
DK-2061	3	70.58096	BOGUI
BOGUI	3	76.61723	DK-2061

Notes:
This report provides multiple comparison tests for all pairwise differences between the means.

DF= Degrees of freedom MSE= Mean Square Error

Table S5. Statistical analysis of the data obtained from activity of α -amylase on flours and purified inhibitors derived from the corn hybrids DK-2061 and BOGUI.

Test of the Normality Distribution

Test Name	Test Value	Prob Level	10% Critical Value	5% Critical Value	Decision (Alpha= 5%)
Kolmogorov-Smirnov	0.192		0.383	0.415	Can't reject normality

Test of the Homoscedasticity

Test Name	Test Value	Prob Level	Reject Equal Variances? (α=0.20)
Levene (Data – Medians)	9.3106	0.00210	Yes

Analysis of Variance Table and F-Test

Model Term	DF	Sum of Squares	Mean Square	F-Ratio	Prob Level	Reject Equal Means? (α=0.05)	Power (α=0.05)
Between (Sample)	4	1037.141	259.2852	52.3320	0.00000	Yes	1.00000
Within (Error)	10	49.54617	4.954617				
Adjusted Total	14	1086.687					
Total	15						

Tukey-Kramer Multiple-Comparison Test

Response: Porcentage_inhibition
Term A: Sample

Alpha=0.050 Error Term=S(A) DF=10 MSE=4.954617 Critical Value=4.6615

Group	Count	Mean	Different From Groups
Flour form hybrid DK-2061	3	31.76665	Flour form hybrid BOGUI, Potato starck<br>Purified inhibitor from DK-2061
Flour form hybrid BOGUI	3	39.50245	Flour form hybrid DK-2061, Potato starck<br>Purified inhibitor from DK-2061<br>Purified inhibitor from BOGUI
Potato starck	3	47.43787	Flour form hybrid DK-2061<br>Flour form hybrid BOGUI<br>Purified inhibitor from DK-2061<br>Purified inhibitor from BOGUI
Purified inhibitor from DK-2061	3	22.65007	Flour form hybrid DK-2061<br>Flour form hybrid BOGUI, Potato starck<br>Purified inhibitor from BOGUI
Purified inhibitor from BOGUI	3	32.13265	Flour form hybrid BOGUI, Potato starck<br>Purified inhibitor from DK-2061

Notes:
This report provides multiple comparison tests for all pairwise differences between the means.

DF= Degrees of freedom MSE= Mean Square Error

Table S6. Statistical analysis of the data obtained in determining the IC50 of the purified inhibitor of the corn hybrid DK-2061 on the activity of the aspartyl protease EAP1 of *S. reilianum*.

Test of the Normality Distribution

Test Name	Test Value	Prob Level	10% Critical Value	5% Critical Value	Decision (Alpha= 5%)
Shapiro-Wilk W	1.000	1.0000			Can't reject normality

Test of the Homoscedasticity

Test Name	Test Value	Prob Level	Reject Equal Variances? (α=0.20)
Levene (Data – Medians)	5.6957	0.00642	Yes

Analysis of Variance Table and F-Test

Model Term	DF	Sum of Squares	Mean Square	F-Ratio	Prob Level	Reject Equal Means? (α=0.05)	Power (α=0.05)
Between (Concentration)	5	18035.01	3607.002	3872.8878	0.00000	Yes	1.00000
Within (Error)	12	11.17616	0.9313468				
Adjusted Total	17	18046.18					
Total	18						

Tukey-Kramer Multiple-Comparison Test

Response: Porcentage_of_inhibition
Term A: Concentration

Alpha=0.050 Error Term=S(A) DF=12 MSE=0.9313468 Critical Value=4.7572

Group	Count	Mean	Different From Groups
0	3	0	5, 10, 15, 20, 25
5	3	11.93167	0, 15, 20, 25
10	3	14.49867	0, 20, 25
15	3	17.05267	0, 5, 20, 25
20	3	51.69133	0, 5, 10, 15, 25
25	3	92.58434	0, 5, 10, 15, 20

Notes:
This report provides multiple comparison tests for all pairwise differences between the means.

DF= Degrees of freedom MSE= Mean Square Error

Table S7. Statistical analysis of the data obtained in determining the IC50 of the purified inhibitor from the corn hybrid BOGUI on the activity of the aspartyl protease EAP1 of *S. reilianum*.

Test of the Normality Distribution

Test Name	Test Value	Prob Level	10% Critical Value	5% Critical Value	Decision (Alpha= 5%)
Shapiro-Wilk W	1.000	1.0000			Can't reject normality

Test of the Homoscedasticity

Test Name	Test Value	Prob Level	Reject Equal Variances? (α=0.20)
Levene (Data – Medians)	3.8619	0.01746	Yes

Analysis of Variance Table and F-Test

Model Term	DF	Sum of Squares	Mean Square	F-Ratio	Prob Level	Reject Equal Means? (α=0.05)	Power (α=0.05)
Between (Concentration)	6	15309.38	2551.563	3187.5560	0.00000	Yes	1.00000
Within (Error)	14	11.20667	0.8004762				
Adjusted Total	20	15320.58					
Total	21						

Tukey-Kramer Multiple-Comparison Test

Response: Porcentage_of_inhibition
Term A: Concentration

Alpha=0.050 Error Term=S(A) DF=14 MSE=0.8004762 Critical Value=4.8356

Group	Count	Mean	Different From Groups
0	3	0	10, 15, 20, 25, 30, 35
10	3	19.96667	0, 15, 20, 25, 30, 35
15	3	26.13333	0, 10, 20, 25, 30, 35
20	3	29.16667	0, 10, 15, 25, 30, 35
25	3	34.5	0, 10, 15, 20, 30, 35
30	3	52.03333	0, 10, 15, 20, 25, 35
35	3	92.1	0, 10, 15, 20, 25, 30

Notes:
This report provides multiple comparison tests for all pairwise differences between the means.

DF= Degrees of freedom MSE= Mean Square Error

Table S8. Statistical analysis of the data obtained in determining the IC50 of the purified inhibitor from the corn hybrid DK-2061 on the activity of the xylanase SRXL1 of *S. reilianum*.

Test of the Normality Distribution

Test Name	Test Value	Prob Level	10% Critical Value	5% Critical Value	Decision (Alpha= 5%)
Shapiro-Wilk W	1.000	1.0000			Can't reject normality

Test of the Homoscedasticity

Test Name	Test Value	Prob Level	Reject Equal Variances? (α=0.20)
Levene (Data – Medians)	4.9421	0.00652	Yes

Analysis of Variance Table and F-Test

Model Term	DF	Sum of Squares	Mean Square	F-Ratio	Prob Level	Reject Equal Means? (α=0.05)	Power (α=0.05)
Between (Concentration)	6	14831.89	2471.981	7005.6145	0.00000	Yes	1.00000
Within (Error)	14	4.94	0.3528571				
Adjusted Total	20	14836.83					
Total	21						

Tukey-Kramer Multiple-Comparison Test

Response: Porcentage_of_inhibition
Term A: Concentration

Alpha=0.050 Error Term=S(A) DF=14 MSE=0.3528571 Critical Value=4.8356

Group	Count	Mean	Different From Groups
0	3	0	2.5, 5, 10, 15, 20, 25
2.5	3	46.4	0, 5, 10, 15, 20, 25
5	3	50.46667	0, 2.5, 10, 15, 20, 25
10	3	54.83333	0, 2.5, 5, 15, 20, 25
15	3	57.5	0, 2.5, 5, 10, 20, 25
20	3	65.66666	0, 2.5, 5, 10, 15, 25
25	3	96.6	0, 2.5, 5, 10, 15, 20

Notes:
This report provides multiple comparison tests for all pairwise differences between the means.

DF= Degrees of freedom MSE= Mean Square Error

Table S9. Statistical analysis of the data obtained in determining the IC50 of the purified inhibitor from the corn hybrid BOGUI on the activity of the xylanase SRXL1 of *S. reilianum*.

Test of the Normality Distribution

Test Name	Test Value	Prob Level	10% Critical Value	5% Critical Value	Decision (Alpha= 5%)
Shapiro-Wilk W	1.000	1.0000			Can't reject normality

Test of the Homoscedasticity

Test Name	Test Value	Prob Level	Reject Equal Variances? (α=0.20)
Levene (Data – Medians)	4.7869	0.02037	Yes

Analysis of Variance Table and F-Test

Model Term	DF	Sum of Squares	Mean Square	F-Ratio	Prob Level	Reject Equal Means? (α=0.05)	Power (α=0.05)
Between (Concentración)	4	14857.8	3714.451	9001.0921	0.00000	Yes	1.00000
Within (Error)	10	4.126667	0.4126667				
Adjusted Total	14	14861.93					
Total	15						

Tukey-Kramer Multiple-Comparison Test

Response: Porcentage_of_inhibition
Term A: Concentration

Alpha=0.050 Error Term=S(A) DF=10 MSE=0.4126667 Critical Value=4.6615

Group	Count	Mean	Different From Groups
0	3	0	2.5, 5, 7.5, 10
2.5	3	42.46667	0, 5, 7.5, 10
5	3	54.33333	0, 2.5, 7.5, 10
7.5	3	64.33334	0, 2.5, 5, 10
10	3	96.73333	0, 2.5, 5, 7.5

Notes:
This report provides multiple comparison tests for all pairwise differences between the means.

DF= Degrees of freedom MSE= Mean Square Error

Table S10. Statistical analysis of data obtained on the effect of the purified inhibitor from the corn hybrid DK-2061 on the V_{max} of the aspartyl protease EAP1 of *S. reilianum*.

Test of the Normality Distribution

Test Name	Test Value	Prob Level	10% Critical Value	5% Critical Value	Decision (Alpha= 5%)
Shapiro-Wilk W	1.000	1.0000			Can't reject normality

Test of the Homoscedasticity

Test Name	Test Value	Prob Level	Reject Equal Variances? (α=0.20)
Levene (Data – Medians)	16.0000	0.00394	Yes

Analysis of Variance Table and F-Test

Model Term	DF	Sum of Squares	Mean Square	F-Ratio	Prob Level	Reject Equal Means? (α=0.05)	Power (α=0.05)
Between (Concentracion_I)	2	0.0005682222	0.0002841111	2557.0000	0.00000	Yes	1.00000
Within (Error)	6	6.666667E-07	1.111111E-07				
Adjusted Total	8	0.0005688889					
Total	9						

Tukey-Kramer Multiple-Comparison Test

Response: Vmax
Term A: Concentration

Alpha=0.050 Error Term=S(A) DF=6 MSE=1.111111E-07 Critical Value=4.3468

Group	Count	Mean	Different From Groups
15	3	0.012	20, No inhibitor
20	3	0.011	15, No inhibitor
No inhibitor	3	0.02833333	15, 20

Notes:
This report provides multiple comparison tests for all pairwise differences between the means.

DF= Degrees of freedom MSE= Mean Square Error

Table S11. Statistical analysis of data obtained on the effect of the purified inhibitor from the corn hybrid BOGUI on the V_{max} of the aspartyl protease EAP1 of *S. reilianum*.

Test of the Normality Distribution

Test Name	Test Value	Prob Level	10% Critical Value	5% Critical Value	Decision (Alpha= 5%)
Shapiro-Wilk W	1.000	1.0000			Can't reject normality

Test of the Homoscedasticity

Test Name	Test Value	Prob Level	Reject Equal Variances? (α=0.20)
Levene (Data – Medians)	1.2983	0.34000	No

Analysis of Variance Table and F-Test

Model Term	DF	Sum of Squares	Mean Square	F-Ratio	Prob Level	Reject Equal Means? (α=0.05)	Power (α=0.05)
Between (Concentration)	2	0.141344	0.070672	155.7796	0.00001	Yes	1.00000
Within (Error)	6	0.002722	0.0004536667				
Adjusted Total	8	0.144066					
Total	9						

Tukey-Kramer Multiple-Comparison Test

Response: Vmax
Term A: Concentration

Alpha=0.050 Error Term=S(A) DF=6 MSE=0.0004536667 Critical Value=4.3468

Group	Count	Mean	Different From Groups
25	3	0.315	30, No inhibitor
30	3	0.379	25, No inhibitor
No inhibitor	3	0.607	25, 30

Notes:
This report provides multiple comparison tests for all pairwise differences between the means.

DF= Degrees of freedom MSE= Mean Square Error

Table S12. Statistical analysis of data obtained on the effect of the purified inhibitor from the corn hybrid DK-2061 on the *Km* of the aspartyl protease EAP1 of *S. reilianum*.

Test of the Normality Distribution

Test Name	Test Value	Prob Level	10% Critical Value	5% Critical Value	Decision (Alpha= 5%)
Kolmogorov-Smirnov	0.279		0.383	0.415	Can't reject normality

Test of the Homoscedasticity

Test Name	Test Value	Prob Level	Reject Equal Variances? (α=0.20)
Levene (Data – Medians)	15.9946	0.00394	Yes

Analysis of Variance Table and F-Test

Model Term	DF	Sum of Squares	Mean Square	F-Ratio	Prob Level	Reject Equal Means? (α=0.05)	Power (α=0.05)
Between (Concentration)	2	12930.7	6465.351	0.9957	0.42324	No	0.15386
Within (Error)	6	38959.46	6493.244				
Adjusted Total	8	51890.17					
Total	9						

Tukey-Kramer Multiple-Comparison Test

Response: Km
Term A: Concentration

Alpha=0.050 Error Term=S(A) DF=6 MSE=6493.244 Critical Value=4.3468

Group	Count	Mean	Different From Groups
15	3	80.83867	
20	3	0.2566667	
No inhibitor	3	0.607	

Notes:
This report provides multiple comparison tests for all pairwise differences between the means.

DF= Degrees of freedom MSE= Mean Square Error

Table S13. Statistical analysis of data obtained on the effect of the purified inhibitor from the corn hybrid BOGUI on the *Km* of the aspartyl protease EAP1 of *S. reilianum*.

Test of the Normality Distribution

Test Name	Test Value	Prob Level	10% Critical Value	5% Critical Value	Decision (Alpha= 5%)
Kolmogorov-Smirnov	0.279		0.383	0.415	Can't reject normality

Test of the Homoscedasticity

Test Name	Test Value	Prob Level	Reject Equal Variances? (α=0.20)
Levene (Data – Medians)	1.2983	0.34000	No

Analysis of Variance Table and F-Test

Model Term	DF	Sum of Squares	Mean Square	F-Ratio	Prob Level	Reject Equal Means? (α=0.05)	Power (α=0.05)
Between (Concentration)	2	0.141344	0.070672	155.7796	0.00001	Yes	1.00000
Within (Error)	6	0.002722	0.0004536667				
Adjusted Total	8	0.144066					
Total	9						

Tukey-Kramer Multiple-Comparison Test

Response: Km
Term A: Concentration

Alpha=0.050 Error Term=S(A) DF=6 MSE=0.0004536667 Critical Value=4.3468

Group	Count	Mean	Different From Groups
25	3	0.315	30, No inhibitor
30	3	0.379	25, No inhibitor
No inhibitor	3	0.607	25, 30

Notes:
This report provides multiple comparison tests for all pairwise differences between the means.

DF= Degrees of freedom MSE= Mean Square Error

Table S14. Statistical analysis of data obtained on the effect of the purified inhibitor from the corn hybrid DK-2061 on the V_{max} of the xylanase SRXL1 of *S. reilianum*.

Test of the Normality Distribution

Test Name	Test Value	Prob Level	10% Critical Value	5% Critical Value	Decision (Alpha= 5%)
Kolmogorov-Smirnov	0.279		0.383	0.415	Can't reject normality

Test of the Homoscedasticity

Test Name	Test Value	Prob Level	Reject Equal Variances? (α=0.20)
Levene (Data – Medians)	2.5641	0.15674	Yes

Analysis of Variance Table and F-Test

Model Term	DF	Sum of Squares	Mean Square	F-Ratio	Prob Level	Reject Equal Means? (α=0.05)	Power (α=0.05)
Between (DK2061)	2	6.612291	3.306146	63.9397	0.00009	Yes	1.00000
Within (Error)	6	0.3102433	0.05170722				
Adjusted Total	8	6.922535					
Total	9						

Tukey-Kramer Multiple-Comparison Test

Response: Vmax
Term A: Concentration

Alpha=0.050 Error Term=S(A) DF=6 MSE=0.05170722 Critical Value=4.3468

Group	Count	Mean	Different From Groups
2.5	3	2.801333	No inhibitor
5	3	2.324	No inhibitor
No inhibitor	3	4.333333	2.5, 5

Notes:
This report provides multiple comparison tests for all pairwise differences between the means.

DF= Degrees of freedom MSE= Mean Square Error

Table S15. Statistical analysis of data obtained on the effect of the purified inhibitor from the corn hybrid BOGUI on the V_{max} of the xylanase SRXL1 of *S. reilianum*.

Test of the Normality Distribution

Test Name	Test Value	Prob Level	10% Critical Value	5% Critical Value	Decision (Alpha= 5%)
Kolmogorov-Smirnov	0.292		0.383	0.415	Can't reject normality

Test of the Homoscedasticity

Test Name	Test Value	Prob Level	Reject Equal Variances? (α=0.20)
Levene (Data – Medians)	1.1082	0.38942	No

Analysis of Variance Table and F-Test

Model Term	DF	Sum of Squares	Mean Square	F-Ratio	Prob Level	Reject Equal Means? (α=0.05)	Power (α=0.05)
Between (Concentration)	2	9.535556	4.767778	158.9259	0.00001	Yes	1.00000
Within (Error)	6	0.18	0.03				
Adjusted Total	8	9.715555					
Total	9						

Tukey-Kramer Multiple-Comparison Test

Response: Vmax
Term A: Concentration

Alpha=0.050 Error Term=S(A) DF=6 MSE=0.03 Critical Value=4.3468

Group	Count	Mean	Different From Groups
2.5	3	3.366667	5 No inhibitor
5	3	1.833333	2.5, No inhibitor
No inhibitor	3	4.333333	2.5, 5

Notes:
This report provides multiple comparison tests for all pairwise differences between the means.

DF= Degrees of freedom MSE= Mean Square Error

Table S16. Statistical analysis of data obtained on the effect of the purified inhibitor from the corn hybrid DK-2061 on the *Km* of the xylanase SRXL1 of *S. reilianum*.

Test of the Normality Distribution

Test Name	Test Value	Prob Level	10% Critical Value	5% Critical Value	Decision (Alpha= 5%)
Kolmogorov-Smirnov	0.000		0.383	0.415	Can't reject normality

Test of the Homoscedasticity

Test Name	Test Value	Prob Level	Reject Equal Variances? (α=0.20)
Levene (Data – Medians)	1.1082	0.38942	No

Analysis of Variance Table and F-Test

Model Term	DF	Sum of Squares	Mean Square	F-Ratio	Prob Level	Reject Equal Means? (α=0.05)	Power (α=0.05)
Between (DK2061)	2	0.228224	0.114112	28.9943	0.00082	Yes	0.99948
Within (Error)	6	0.023614	0.003935667				
Adjusted Total	8	0.251838					
Total	9						

Tukey-Kramer Multiple-Comparison Test

Response: Km
Term A: Concentration

Alpha=0.050 Error Term=S(A) DF=6 MSE=0.003935667 Critical Value=4.3468

Group	Count	Mean	Different From Groups
2.5	3	0.7706667	No inhibitor
5	3	0.6986667	No inhibitor
No inhibitor	3	1.066667	2.5, 5

Notes:
This report provides multiple comparison tests for all pairwise differences between the means.

DF= Degrees of freedom MSE= Mean Square Error

Table S17. Statistical analysis of data obtained on the effect of the purified inhibitor from the corn hybrid BOGUI on the K_m of the xylanase SRXL1 of *S. reilianum*.

Test of the Normality Distribution

Test Name	Test Value	Prob Level	10% Critical Value	5% Critical Value	Decision (Alpha= 5%)
Kolmogorov-Smirnov	0.385		0.383	0.415	Can't reject normality

Test of the Homoscedasticity

Test Name	Test Value	Prob Level	Reject Equal Variances? (α=0.20)
Levene (Data – Medians)	0.0000	1.00000	No

Analysis of Variance Table and F-Test

Model Term	DF	Sum of Squares	Mean Square	F-Ratio	Prob Level	Reject Equal Means? (α=0.05)	Power (α=0.05)
Between (Concentration)	2	0.4022222	0.2011111	60.3333	0.00011	Yes	1.00000
Within (Error)	6	0.02	0.003333333				
Adjusted Total	8	0.4222222					
Total	9						

Tukey-Kramer Multiple-Comparison Test

Response: Km
Term A: Concentration

Alpha=0.050 Error Term=S(A) DF=6 MSE=0.003333333 Critical Value=4.3468

Group	Count	Mean	Different From Groups
2.5	3	0.9333333	5
5	3	0.5666667	2.5, No inhibitor
No inhibitor	3	1.066667	5

Notes:
This report provides multiple comparison tests for all pairwise differences between the means.

DF= Degrees of freedom MSE= Mean Square Error
